# Review of AlphaFold 3: Transformative Advances in Drug Design and Therapeutics

**DOI:** 10.7759/cureus.63646

**Published:** 2024-07-02

**Authors:** Dev Desai, Shiv V Kantliwala, Jyothi Vybhavi, Renju Ravi, Harshkumar Patel, Jitendra Patel

**Affiliations:** 1 Research, Albert Einstein College of Medicine, New York, USA; 2 Medicine, Smt. Nathiba Hargovandas Lakhmichand Municipal Medical College, Ahmedabad, IND; 3 Public Health, Griffith University, Brisbane, AUS; 4 Physiology, RajaRajeswari Medical College and Hospital, Bangalore, IND; 5 Clinical Pharmacology, Faculty of Medicine, Jazan University, Jizan, SAU; 6 Internal Medicine, Gujarat Medical Education and Research Society Medical College, Vadnagar, IND; 7 Physiology, Gujarat Medical Education and Research Society Medical College, Vadnagar, IND

**Keywords:** google deepmind, predictive biochemistry, artificial intelligence, protein structure, structural biology, deep learning, machine learning, deepmind, alphafold 3

## Abstract

Google DeepMind Technologies Limited (London, United Kingdom) recently released its new version of the biomolecular structure predictor artificial intelligence (AI) model named AlphaFold 3. Superior in accuracy and more powerful than its predecessor AlphaFold 2, this innovation has astonished the world with its capacity and speed. It takes humans years to determine the structure of various proteins and how the shape works with the receptors but AlphaFold 3 predicts the same structure in seconds. The version's utility is unimaginable in the field of drug discoveries, vaccines, enzymatic processes, and determining the rate and effect of different biological processes. AlphaFold 3 uses similar machine learning and deep learning models such as Gemini (Google DeepMind Technologies Limited). AlphaFold 3 has already established itself as a turning point in the field of computational biochemistry and drug development along with receptor modulation and biomolecular development. With the help of AlphaFold 3 and models similar to this, researchers will gain unparalleled insights into the structural dynamics of proteins and their interactions, opening up new avenues for scientists and doctors to exploit for the benefit of the patient. The integration of AI models like AlphaFold 3, bolstered by rigorous validation against high-standard research publications, is set to catalyze further innovations and offer a glimpse into the future of biomedicine.

## Introduction and background

On May 8, 2024, an article was published by Josh Abramson regarding the successful use of AlphaFold 3 (Google DeepMind Technologies Limited, London, United Kingdom) in the accurate structure prediction of biomolecular interactions [1].

In the rapidly evolving landscape of life sciences, the intersection of artificial intelligence (AI) and biology has heralded a new era of discovery and innovation. At the forefront of this revolution is AlphaFold 3, the latest iteration of Google DeepMind's groundbreaking protein folding AI. Leveraging advanced deep learning methodologies, AlphaFold 3 has achieved unprecedented accuracy in predicting the three-dimensional structures of proteins, a cornerstone in understanding biomolecular interactions and functions [2]. This capability not only enhances our grasp of biological machinery but also accelerates the pace at which we can address some of the most pressing challenges in medical biology, from antibiotic resistance to the development of malaria vaccines [1,3,4].

This review, by exploring advancements in machine learning and deep learning technologies such as the the innovative use of AI by Bileschi and Colwell [5] to harness molecular structure predictions and the Gemini model (Google DeepMind Technologies Limited) [6], will attempt to reveal how AlphaFold 3 sets new benchmarks in the accurate modeling of protein structures and their complexes with ligands. Further, we will explore its applications in drug discovery, providing real-life case studies and examples that underscore its potential. Alongside the success stories, we will also address the challenges and limitations faced by researchers, setting the stage for a discussion on the prospects and developments in leveraging AI, like AlphaFold 3, for breakthroughs in the life sciences [1,7,8].

## Review

Overview of AlphaFold 3 and its capabilities

AlphaFold 3, developed by Isomorphic Labs Limited (London, United Kingdom) and Google DeepMind, represents a significant leap forward in the field of molecular biology through its ability to predict the structure and interactions of biomolecules with unparalleled accuracy [6]. This revolutionary model extends its predictive capabilities beyond proteins to encompass a broad spectrum of biomolecules, including proteins, DNA, RNA, and ligands, thereby offering a comprehensive understanding of the biological world.

Predictive Accuracy and Methodology

AlphaFold 3's predictive prowess is rooted in its next-generation architecture, which includes an improved version of the Evoformer module [6]. This deep learning architecture was instrumental in the success of its predecessor, AlphaFold 2, and has been further refined to enhance performance. The model employs a diffusion network process, starting with a cloud of atoms and iteratively converging on the most accurate molecular structure. This methodology allows AlphaFold 3 to generate joint three-dimensional (3D) structures of input molecules, revealing how they fit together holistically [1].

Expanded Capabilities

One of the most notable advancements of AlphaFold 3 is its expanded capabilities to model large biomolecules such as proteins, DNA, and RNA, as well as small molecules known as ligands. This expansion is crucial for drug discovery as it includes many drugs under the ligand category. Additionally, AlphaFold 3 can model chemical modifications to these molecules, which play a significant role in the healthy functioning of cells. Disruptions in these processes can lead to disease, thus highlighting the model's potential in understanding and addressing medical conditions [9-11].

Impact on Drug Discovery and Biological Understanding

The introduction of AlphaFold 3 has the potential to transform drug discovery by providing a more rapid and accurate tool for examining fundamental biology [4]. Its ability to predict the structure of protein-molecule complexes, including those containing DNA and RNA, marks a significant improvement over existing prediction methods. This capability is especially valuable for drug discovery, as it aids in identifying and designing new molecules that could lead to effective treatments. Researchers are excited about the prospect of speedier drug discovery, facilitated by the greater range of predictive abilities offered by AlphaFold 3 [1,6,12,13].

Accessibility and Collaborative Efforts

To maximize the impact of AlphaFold 3 on scientific research and drug discovery, its capabilities are made accessible to scientists through the AlphaFold Server. This easy-to-use research tool allows the scientific community to leverage the model's predictive abilities free. Furthermore, Isomorphic Labs is collaborating with pharmaceutical companies to apply AlphaFold 3 to real-world drug design challenges, aiming to develop new treatments that could change patients' lives. This collaborative approach underscores the commitment to harnessing the potential of AlphaFold 3 for advancing medical science and research.

In summary, AlphaFold 3's introduction marks a pivotal moment in the understanding of biomolecular interactions and the acceleration of drug discovery processes. Its comprehensive modeling capabilities, combined with its predictive accuracy, set a new standard in the field, promising to unlock transformative scientific advancements [1].

Understanding biomolecular interactions

AlphaFold 3 stands as a monumental advancement in the realm of computational biology, offering unprecedented insights into the intricate dance of biomolecules that underpin life itself. This section delves into the core of AlphaFold 3's capabilities, shedding light on its profound impact on our understanding of biomolecular interactions [11,14].

Predictive Power Across the Biomolecular Spectrum

AlphaFold 3's methodology allows for the generation of joint 3D structures of molecules, facilitating a comprehensive view of how various biomolecules, including proteins, DNA, RNA, and ligands, interact within a cellular context. This capability is crucial for drug discovery, as many drugs fall under the ligand category. Moreover, AlphaFold 3's ability to model chemical modifications to these molecules provides insights into cellular processes essential for health, the disruption of which can lead to disease.

Unprecedented Accuracy in Molecular Interactions

The model's predictive accuracy in modeling molecular interactions, including the binding of proteins with ligands and antibodies with their target proteins, sets a new standard. AlphaFold 3 is reported to be 50% more accurate than the best traditional methods on the PoseBusters benchmark, marking it as the first AI system to outperform physics-based tools in biomolecular structure prediction [6]. This leap in predictive accuracy offers a new lens through which to view the molecular underpinnings of life, from drug interactions to the human immune response.

Expanding the Frontier of Drug Discovery

AlphaFold 3's nuanced understanding of protein-ligand interactions holds immense potential for revolutionizing drug discovery [15]. By accurately predicting the binding sites and optimal shapes for potential drug molecules, AlphaFold 3 significantly streamlines the drug design process. This efficiency could drastically reduce the time and cost associated with experimental methods, allowing researchers to focus on the most promising drug candidates.

Integrating Structural Predictions with Genomic Insights

While AlphaFold 3 brings unparalleled precision to protein structure prediction, its utility in drug discovery is complemented by tools like Cognit (Cognit AI, San Francisco, California, United States), which focus on the genomic and transcriptomic foundations of health and disease. The integration of Cognit's upstream genomic and transcriptomic insights with AlphaFold 3's structural predictions creates a powerful synergy in drug discovery [16]. This holistic approach covers the entire spectrum of drug development, from early-stage target discovery and validation to the optimization of therapeutic interactions at the molecular level.

AlphaFold 3's contributions to our understanding of biomolecular interactions offer a new window into the molecular dynamics that drive life's processes. By providing detailed insights into how molecules interact and influence biological functions, it paves the way for groundbreaking advancements in medical science and research [17].

Advancements over previous versions

Enhanced Architectural Framework

AlphaFold 3 represents a significant evolution from its predecessor, AlphaFold 2, primarily through its advanced architectural framework [1,9,10,18]. The core of this new model, the Evoformer module, has been significantly enhanced to improve performance across a broader spectrum of biomolecules, extending beyond proteins to include DNA, RNA, and ligands [1]. This architectural refinement has enabled AlphaFold 3 to achieve unprecedented accuracy in predicting drug-like interactions, setting a new benchmark in the field [6].

Integration of Novel Neural Network Architectures

The introduction of novel neural network architectures in AlphaFold 3 has been pivotal in enhancing its predictive capabilities [19]. These innovations include a scaled-down MSA processing unit and the new "Pairformer," which focuses solely on pair and single representations, eliminating the need for MSA representation. This simplification allows for a more focused and efficient prediction process. Additionally, the diffusion module, a new addition to AlphaFold 3, directly handles raw atom coordinates, streamlining the prediction process by eliminating the need for complex rotational adjustments [1,4,15].

Iterative Refinement and Accuracy

AlphaFold 3 utilizes a unique iterative refinement process, termed "recycling", which significantly contributes to its enhanced accuracy. This process involves the repeated application of the final loss to outputs, which are then recursively fed back into the network [19]. This method allows for continuous refinement and development of highly accurate protein structures with precise atomic details. The structure module has also been redesigned to include an explicit 3D structure for each residue, rapidly developing and refining a highly accurate protein structure [18].

Expanded Predictive Abilities

One of the most notable advancements in AlphaFold 3 is its expanded predictive abilities. The model now accurately predicts protein-molecule complexes that include a variety of biomolecules such as DNA and RNA. This expansion is crucial for applications in genomics research and drug discovery, providing researchers with a powerful tool for faster and more accurate predictions. The model's ability to predict complex interactions without needing input structural information marks it as the first AI system to surpass traditional physics-based tools in biomolecular structure prediction [6].

These advancements position AlphaFold 3 as a transformative tool in the field of computational biology, offering deeper insights and more accurate predictions that pave the way for innovative solutions in medical science and beyond.

Impact on medical science and research

Transformative Advances in Drug Design and Therapeutics

AlphaFold 3 has significantly enhanced the capabilities for drug design, particularly in the accurate prediction of molecular interactions crucial for therapeutic development. By modeling proteins, ligands, and antibodies with unprecedented precision, this AI-driven tool allows for the rapid design of drugs targeting specific molecules, which is especially vital in cancer research [8]. The ability to design molecules that can specifically bind to target proteins may lead to more effective treatments with fewer side effects, revolutionizing therapeutic approaches [20-24].

Accelerating Discovery and Reducing Development Costs

The predictive power of AlphaFold 3 extends beyond traditional methods like X-ray crystallography or cryo-electron microscopy, which are both time-consuming and costly. AlphaFold 3 can predict complex molecular structures in a fraction of the time, dramatically reducing both the time and financial costs associated with drug development [8]. This efficiency allows researchers to focus on the most promising drug targets and biological questions without the burden of lengthy and expensive traditional methods [20,21].

Enhancing Understanding of Immune Responses

AlphaFold 3's ability to predict interactions between the spike protein of common viruses and antibodies provides critical insights into immune response mechanisms. This capability is instrumental in advancing our understanding of how diseases like COVID-19 can be combated through the immune system, potentially leading to more effective treatments and vaccines [23,24]. By modeling these interactions with high accuracy, AlphaFold 3 aids in the development of therapeutic strategies that are finely tuned to the nuances of immune system behavior [8].

Broadening the Scope of Scientific Inquiry

With its comprehensive approach to modeling life’s molecules, AlphaFold 3 broadens the scope of scientific inquiry, enabling researchers to explore a wider range of biological molecules and their interactions [12,22]. This expansion of capabilities accelerates the discovery process and opens new avenues for research that were previously limited by technological constraints. Insights gained from these studies could lead to breakthroughs in various fields, including the development of more resilient crops and novel therapeutic proteins and antibodies [22-24].

Pioneering New Treatments with AI Integration

By integrating AlphaFold 3 with other AI models and tools, Isomorphic Labs is pioneering new treatments and approaches to drug design. This collaborative effort leverages the enhanced predictive abilities of AlphaFold 3 to tackle disease targets that were previously challenging to address, paving the way for the development of innovative treatments. The use of AlphaFold 3 in these efforts exemplifies the potential of AI to transform medical science and research by providing deeper insights and more accurate predictions than ever before [24].

AlphaFold 3 brings new possibilities for understanding complex biological interactions and developing effective therapeutic interventions. Its capabilities not only enhance the efficiency of research but also promise to usher in a new era of precision medicine, driven by deep insights into the molecular foundations of health and disease [1,4,20-24].

Applications in drug discovery

Streamlining Drug Candidate Identification

Machine learning, particularly through platforms like AlphaFold 3, has revolutionized the identification and optimization of lead compounds in drug discovery [25,26]. By analyzing extensive chemical libraries, AlphaFold 3 identifies promising drug candidates based on their predicted biological activity and physicochemical properties, significantly accelerating the drug development process.

Enhancing Drug Design with Predictive Accuracy

AlphaFold 3's ability to predict drug-target interactions with unprecedented accuracy is a cornerstone of its application in drug discovery. This AI model predicts how new drug candidates will interact with biological targets, aiding in the design of more effective and selective drugs. Its predictive prowess is 50% more accurate than traditional methods, making it a valuable tool for developing new therapeutics [6,20].

Facilitating Drug Repurposing

The use of machine learning in drug repurposing has become increasingly important. AlphaFold 3 analyzes data on drug-target interactions and side effects to identify new therapeutic applications for existing drugs. This approach not only saves time and resources but also offers a path to rediscover the potential of existing pharmacological agents [23,27].

*Predicting ADME (Absorption, Distribution, Metabolism, and Excretion)* *Properties*

AlphaFold 3 extends its utility to predicting ADME properties, which are crucial for understanding a drug's performance within the body. This capability supports the optimization of clinical trial designs by predicting how drugs behave in different biological systems, thereby improving the safety and efficacy of therapeutic candidates [26,28,29].

Personalized Medicine Approaches

By enabling the rapid design and customization of drugs based on individual protein structures, AlphaFold 3 opens doors to personalized medicine. This approach tailors treatments to individual genetic profiles, enhancing treatment efficacy and reducing side effects, thereby transforming patient care in significant ways.

Collaborative Drug Design Initiatives

Isomorphic Labs leverages AlphaFold 3 in collaboration with pharmaceutical partners to enhance drug design processes [6]. This partnership approach not only accelerates drug development cycles but also ensures that new treatments are both innovative and effective, addressing previously intractable disease targets.

Cost Reduction in Drug Development

The predictive capabilities of AlphaFold 3 reduce the necessity for extensive wet lab experiments, thereby saving millions in development costs. By accurately predicting drug interactions and potential toxicity, AlphaFold 3 minimizes the progression of ineffective candidates into clinical trials, significantly cutting down on development time and expense [21,23,26].

Novel Approaches in Target Discovery

AlphaFold 3's expanded predictive abilities include interactions with DNA, RNA, and small molecules, crucial for understanding most disease mechanisms [1]. This broadened scope allows for the identification of new drug targets and the optimization of lead compounds, further enhancing the efficiency and scope of drug discovery efforts.

By integrating these advanced predictive tools, AlphaFold 3 not only streamlines the drug discovery process but also opens up new possibilities for innovative therapeutic approaches, ultimately aiming to improve patient outcomes and advance the field of medical science.

Real-life case studies and examples

AlphaFold in Viral Research and Phage Therapy

AlphaFold's capabilities extend into the realm of viral research, significantly impacting the development of therapies. For instance, its computational predictions have advanced the understanding of bacteriophage receptor-binding proteins, which are crucial for effective phage therapy. These predictions facilitate the discovery of enzymes originating from bacteriophages that can degrade bacterial pathogens' cell walls, offering new avenues in antibacterial treatment strategies [21,30,31].

Enhancing Vaccine Development

In the fight against viruses like SARS-CoV-2, AlphaFold has been instrumental. The platform has aided in designing monomeric receptor-binding domain derivatives for vaccines, showing a superior immune response in mice, a step forward in vaccine development. This example highlights how predictive models can optimize vaccine formulations to enhance their effectiveness [21,32-34].

Drug Discovery in Viral Diseases

AlphaFold's impact is notable in drug discovery, particularly for viral diseases. The platform has been used to identify potential inhibitors for the non-structural protein 6 (NSP6) of SARS-CoV-2, with candidates recommended for biological testing. This approach not only accelerates the identification of viable drug candidates but also refines the drug development process by focusing on the most promising targets [35].

Structural Insights into Complex Viral Proteins

The accuracy of AlphaFold in predicting complex viral protein structures is revolutionary. It has provided near-perfect matches for structures like the spike protein of a cold virus interacting with antibodies and simple sugars [6]. Such detailed structural insights are invaluable for understanding the mechanisms of viral infection and aiding in the development of targeted therapies.

Contributions to Understanding Enzymatic Functions in Agriculture

AlphaFold has also made significant contributions outside of human medicine, such as in agriculture. By predicting the structure of enzymes from soil-borne fungi that affect plant health, researchers can develop strategies to enhance crop resilience and health. This application demonstrates the versatility of AlphaFold in contributing to various scientific fields beyond human health [1,36].

These real-life examples underscore the transformative potential of AlphaFold in advancing our understanding of complex biological interactions and accelerating the pace of medical and scientific discovery. The integration of this technology into various research and development sectors marks a significant leap forward in how we approach and solve biological and medical challenges.

Challenges and limitations

Accuracy and Reliability Concerns

AlphaFold 3, despite its advancements, exhibits varying accuracy levels across different biomolecular interactions. Success rates fluctuate significantly, ranging from 40% to over 80%, with notable inaccuracies in protein-RNA interactions. This variability underscores the necessity for continued refinement and validation of the model's predictions in diverse biological scenarios [1,4,16].

Risk of Hallucination in Structural Predictions

The innovative use of diffusion techniques in AlphaFold 3 introduces a risk of "hallucination," where the model might generate plausible but non-existent molecular structures. Efforts to add more training data have been made, yet this issue has not been fully resolved, posing a challenge in relying solely on AI predictions without further empirical verification [1].

Restricted Access and Its Implications

Unlike its predecessor, AlphaFold 2, the full code of AlphaFold 3 is not publicly available. Access is provided through the AlphaFold Server, which is limited to non-commercial use and restricts the types of molecules that can be studied. This limitation hampers the ability of the broader scientific community to experiment, verify, and potentially improve upon the mode [10,18].

Impact of Access Limitations on Scientific Innovation

The decision to restrict access to AlphaFold 3's full capabilities could potentially stifle innovation and broader application within the scientific community. Limiting the model's use to non-commercial purposes and controlling the types of molecules that can be studied may slow down the pace of discovery and application, especially in environments where commercial use could accelerate advancements [9,25,26].

Challenges in Translating Predictions to Clinical Success

Translating the predictive insights provided by AlphaFold 3 into successful clinical outcomes remains a significant challenge. The complexity of drug interactions and physiological responses often requires more than accurate structural predictions to achieve effective therapeutic results [24,37].

Equity and Transparency in Access to Technology

The limited and proprietary nature of AlphaFold's accessibility raises concerns regarding equitable access to cutting-edge technology. Such restrictions can impact scientific transparency and public accountability, which are crucial for the advancement and democratization of scientific research [38].

By addressing these challenges and limitations, the potential of AlphaFold 3 in transforming medical science and research can be more fully realized, ensuring that this revolutionary technology benefits a broader spectrum of the scientific community.

Future prospects and developments

Expanding Horizons in Drug Discovery

AlphaFold 3's integration into drug discovery processes signifies a transformative shift, particularly with its ability to predict interactions at the atomic level. This precision facilitates the development of drugs that are not only more effective but also specifically targeted, reducing the time and costs associated with traditional drug development methods.

Advancements in Genomics and Personalized Medicine

The detailed structural insights provided by AlphaFold 3 into DNA and RNA open new avenues in genomics, significantly impacting personalized medicine. By understanding how genetic variations affect protein functions, tailored therapeutic strategies can be developed for individuals based on their unique genetic profiles [1].

Innovations in Biomaterials and Bioengineering

AlphaFold 3's capability to predict the structure of small molecules paves the way for creating novel biomaterials. These materials could be specifically designed for biocompatibility and functionality, ranging from medical implants to bioengineered enzymes, which could revolutionize multiple industries.

Fundamental Biological Research

The tool enhances fundamental biological research by providing a deeper understanding of protein folding, cellular signaling, and gene regulation. Such insights lay the groundwork for pioneering discoveries across various biological fields, potentially leading to breakthroughs in understanding complex life processes [1].

Future Developments in AI and Molecular Modeling

Continued advancements in AI, as seen with the introduction of AlphaFold 3, promise further enhancements in biomolecular modeling. The integration of new neural network architectures and iterative refinement processes is expected to improve the accuracy and efficiency of these models, broadening their applicability in scientific research.

Collaborative and Open-Source Efforts

Despite current restrictions on access to AlphaFold 3, there is potential for the development of open-source versions by the end of the year [3]. Such efforts would democratize access to this advanced technology, allowing a broader scientific community to contribute to and benefit from its capabilities.

AlphaFold 3 stands at the forefront of a scientific revolution, with its impact extending beyond immediate applications in drug discovery and genomics to potentially reshape our approach to biological research and material science. As this technology evolves, its integration into various scientific domains is likely to accelerate discoveries and innovations, marking a new era in the intersection of AI and biology [1].

## Conclusions

AlphaFold 3, an AI model, has revolutionized medical biology, computational biochemistry, and drug discovery by providing insights into protein structural dynamics and interactions. This model offers a robust platform for accelerating therapeutic innovations and enhancing our understanding of life's molecular underpinnings. The implications of AlphaFold 3 extend beyond immediate applications, redefining medical challenges, streamlining drug development, and paving the way for personalized medicine. The collaborative ethos and potential broadening of access to AlphaFold 3 signal a democratization of high-level computational tools, empowering diverse research initiatives. The integration of AI models like AlphaFold 3 will catalyze further innovations, offering a glimpse into the future of biomedicine.
